# Subcutaneous hematoma due to submental liposuction: a case report

**DOI:** 10.1093/jscr/rjaf1070

**Published:** 2026-01-31

**Authors:** Zia Obeidavi, Mahshid Garmsiri

**Affiliations:** Aesthetic Medicine Researcher, Private practice, Ahvaz, Khouzestan Province 6155690011, Iran; Aesthetic Medicine Researcher, Private practice, Ahvaz, Khouzestan Province 6155690011, Iran

**Keywords:** liposuction, subcutaneous fat, hematoma, airway obstruction

## Abstract

A young woman developed a large hematoma after submental liposuction, causing swallowing and breathing issues. The patient reported shortness of breath (SOB), along with neck pain, swelling, bruising, and reduced range of motion (ROM). Lab tests showed elevated leukocytes [14.1 × 10^3^/μL (4.50–10.00), normal hemoglobin (12 g/dL (12–16 g/dL), normal platelets (270 × 10^3^ U/L (150–450 × U/L)], and positive CRP. Treatment included antibiotics, hydrocortisone, and clot removal. Regular examinations showed significant improvement without lesions. By the 10th day, edema had completely resolved, with marked improvement in bruising, respiratory symptoms, and neck mobility. Thorough evaluation of candidates for submental liposuction is vital. A doctor’s expertise in techniques and anatomical knowledge can prevent vascular damage. Timely diagnosis and intervention can effectively manage complications and enhance liposuction results.

## Introduction

Previous studies show increased submental fat reduces perceived attractiveness [1–3], leading to more interventions to reduce it. Among the non-surgical measures, we can mention the use of injectable deoxycholic acid, cryolipolysis, devices based on ultrasound waves, but in contrast, the gold standard of fat removal in the submental area is liposuction, which in addition to removing fat in this area causes facial contouring is also done [4]. Liposuction of submental fat is performed under local anesthesia or general anesthesia. Complications caused by submental liposuction are rare, but the lack of accurate knowledge of these complications or how to manage them can have irreparable consequences. Among these complications, hematoma can lead to airway obstruction if not promptly diagnosis or properly managed [5–7]. In this article, we will introduce a young woman who suffered from a large hematoma after liposuction in the submental area with swallowing and breathing disorders.

## Case report

**Figure 1:**
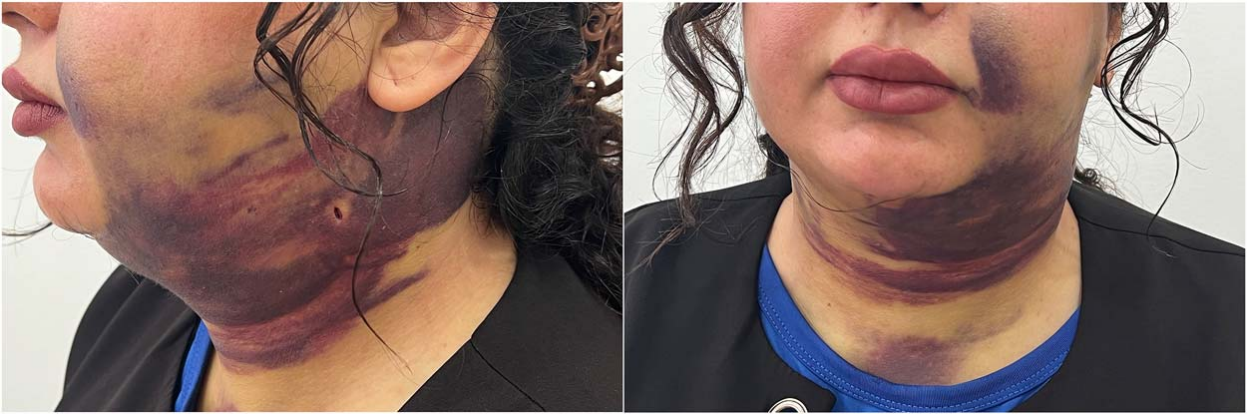
Left lateral and frontal views showing diffuse ecchymosis and swelling in the submental and anterior neck regions extending to the upper chest, four days after submental liposuction.

**Figure 2:**
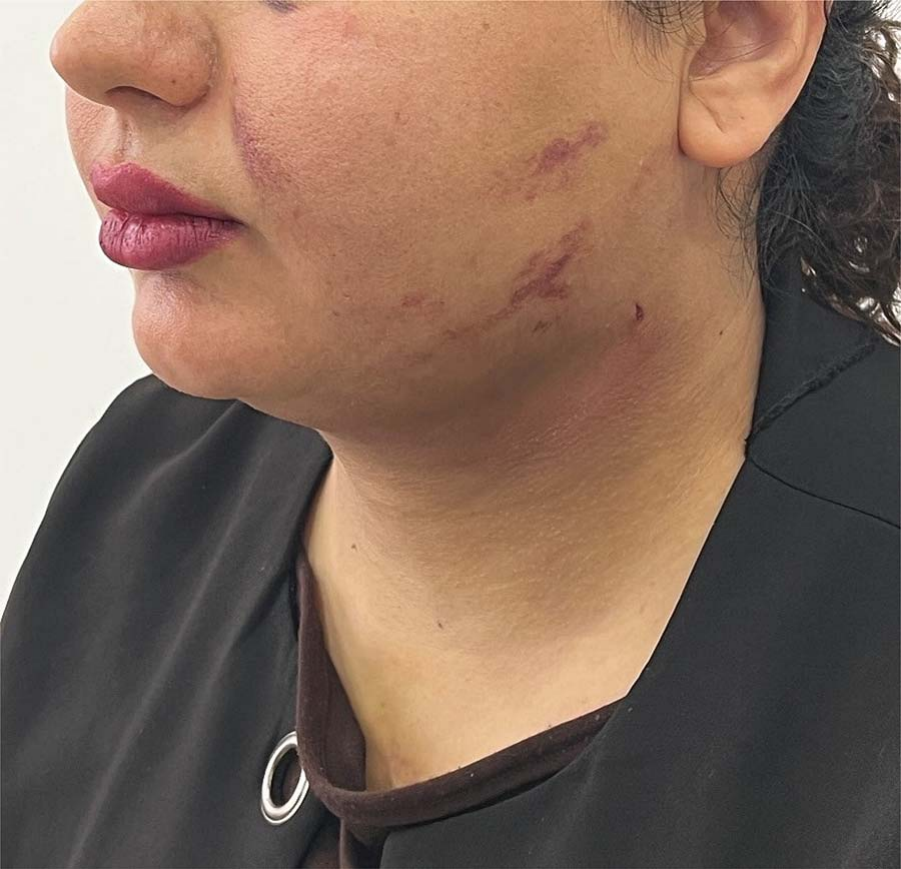
Resolution of swelling and marked reduction of ecchymosis by day 10 post-treatment.

The patient is a 25-year-old young woman who was referred to the medical center four days after submental liposuction due to swallowing and breathing disorders and severe neck pain. On examination, the patients was alert, oriented, afebrile, and without respiratory distress. Vital signs at presentation were as follows: blood pressure, 110/70 mmHg; heart rate, 89 beats per minute; respiratory rate, 22 breaths per minute. The patient did not have a fever and complained of limited neck movement, shortness of breath, pain, extensive bruises, and a swollen appearance of the neck (Fig. 1) after liposuction. She was on oral antibiotics as prescribed. In the initial examinations, swelling of the anterior triangle of the left side of the neck (including the submental, submandibular, carotid, and muscular triangles) with extension to the left occipital triangle and the lower part of the face on the left side with ecchymosis was evident. The site of the liposuction incisions was free of any bleeding or discharge. Other examinations and tests showed no abnormal findings. Based on the initial evaluations and considering the patient’s condition and the limitation of radiological imaging, the patient was diagnosed with subcutaneous hematoma. No ultrasound examination was performed due to limited imaging availability; however, the diagnosis of subcutaneous hematoma was confirmed clinically and by aspiration of dense subcutaneous clots consistent with hematoma. Following the diagnosis of subcutaneous hematoma, treatment was initiated with the administration of 100 mg of hydrocortisone and subsequent antibiotic therapy. In addition, after the injection of 2% lidocaine using a five cc syringe and an 18-gauge needle with a length of 30 mm, the accumulated clots were removed. Approximately 25 mL of dense subcutaneous clots were aspirated cumulatively over five consecutive drainage sessions. She refused hospitalization, so daily clot drainage was performed for five days. We examined the patient at regular intervals, and based on these periodic assessments, we observed considerable improvement with no remaining lesions. By the 10th day, edema had completely resolved, with marked improvement in bruising, respiratory symptoms, and neck mobility (Fig. 2).

## Discussion

Submental liposuction enables removal of excess anterior cervical fat and improves facial contour through the use of a cannula introduced via one- to two-millimeter incisions placed along the mandibular border [1–3]. It can be performed under general or local anesthesia [4]. Complications associated with submental liposuction are rare. Despite this issue, in case of complications, it is essential to correctly and timely diagnose and take appropriate measures [5]. Among these complications, we can mention hematoma. Hematoma can lead to airway obstruction and, if diagnosed late, the death of the patient [5, 6]. Based on the results of a review study conducted by Alves Diniz and colleagues in 2022, which examined the complications associated with submental liposuction, hematoma formation was the main risk associated with this procedure, which may result in skin necrosis [4]. Predisposing factors include vessel injury, no garment use, or poor drainage. Hematoma may also cause necrosis, infection, or deformity [8]. In the referring patient, the first step was to drain the accumulated clots under the skin, which led to the improvement of the patient’s dysphagia, breathing, and dysphonia, as well as the improvement of his restlessness [4, 9, 10]. Because corticosteroids have anti-inflammatory properties that can induce a prothrombotic effect, we administered a dose of hydrocortisone. However, its effectiveness in improving submental hematoma still requires further investigation. In addition, the patient received antibiotics to prevent infection. In the end, it seems necessary to mention a few points. First, the correct evaluation of candidates for submental liposuction before any procedure can be helpful for the necessity of performing liposuction as well as the risk factors related to the high probability of bleeding following any procedure. For example, Baker *et al.* reported that preoperative blood pressure above 150/100 mmHg increases the risk of hematoma by 2.6 times in patients undergoing submental liposuction [11, 12]. In addition, the doctor’s mastery of correct techniques and accurate knowledge of the anatomy of the liposuction area can prevent vascular damage in many cases. Leaving a thin layer of subcutaneous fat during liposuction can help preserve the subdermal vascular network and reduce the risk of hematoma and other complications. In addition, timely diagnosis and correct action can lead to proper management of the complications and improvement of liposuction results.
